# Intraperitoneal chemotherapy for peritoneal metastases of gastric origin: a systematic review and meta-analysis

**DOI:** 10.1093/bjs/znae116

**Published:** 2024-05-09

**Authors:** Niels A D Guchelaar, Kazem Nasserinejad, Bianca Mostert, Stijn L W Koolen, Pieter C van der Sluis, Sjoerd M Lagarde, Bas P L Wijnhoven, Ron H J Mathijssen, Bo J Noordman

**Affiliations:** Department of Medical Oncology, Erasmus Medical Center Cancer Institute, Rotterdam, The Netherlands; Department of Hematology, Erasmus Medical Center Cancer Institute, Rotterdam, The Netherlands; HOVON Foundation, Rotterdam, The Netherlands; Department of Medical Oncology, Erasmus Medical Center Cancer Institute, Rotterdam, The Netherlands; Department of Medical Oncology, Erasmus Medical Center Cancer Institute, Rotterdam, The Netherlands; Department of Pharmacy, Erasmus Medical Center, Rotterdam, The Netherlands; Department of Surgery, Division of Surgical Oncology and Gastrointestinal Surgery, Erasmus Medical Center Cancer Institute, Rotterdam, The Netherlands; Department of Surgery, Division of Surgical Oncology and Gastrointestinal Surgery, Erasmus Medical Center Cancer Institute, Rotterdam, The Netherlands; Department of Surgery, Division of Surgical Oncology and Gastrointestinal Surgery, Erasmus Medical Center Cancer Institute, Rotterdam, The Netherlands; Department of Surgery, Division of Surgical Oncology and Gastrointestinal Surgery, Erasmus Medical Center Cancer Institute, Rotterdam, The Netherlands; Department of Surgery, Division of Surgical Oncology and Gastrointestinal Surgery, Erasmus Medical Center Cancer Institute, Rotterdam, The Netherlands

## Abstract

**Background:**

Gastric cancer with peritoneal metastases is associated with a dismal prognosis. Normothermic catheter-based intraperitoneal chemotherapy and normothermic pressurized intraperitoneal aerosol chemotherapy (PIPAC) are methods to deliver chemotherapy intraperitoneally leading to higher intraperitoneal concentrations of cytotoxic drugs compared to intravenous administration. We reviewed the effectiveness and safety of different methods of palliative intraperitoneal chemotherapy.

**Methods:**

Embase, MEDLINE, Web of Science and Cochrane were searched for articles studying the use of repeated administration of palliative intraperitoneal chemotherapy in patients with gastric cancer and peritoneal metastases, published up to January 2024. The primary outcome was overall survival.

**Results:**

Twenty-three studies were included, representing a total of 999 patients. The pooled median overall survival was 14.5 months. The pooled hazard ratio of the two RCTs using intraperitoneal paclitaxel and docetaxel favoured the intraperitoneal chemotherapy arm. The median overall survival of intraperitoneal paclitaxel, intraperitoneal docetaxel and PIPAC with cisplatin and doxorubicin were respectively 18.4 months, 13.2 months and 9.0 months. All treatment methods had a relatively safe toxicity profile. Conversion surgery after completion of intraperitoneal therapy was performed in 16% of the patients.

**Conclusions:**

Repeated intraperitoneal chemotherapy, regardless of method of administration, is safe for patients with gastric cancer and peritoneal metastases. Conversion surgery after completion of the intraperitoneal chemotherapy is possible in a subset of patients.

## Teaser

This study shows that repeated intraperitoneal chemotherapy, regardless of method of administration, is a promising and safe treatment for patients with gastric cancer and peritoneal metastases. Conversion surgery after completion of the intraperitoneal chemotherapy is possible in a selected subset of patients.

## Introduction

Gastric cancer is the fourth leading cause of cancer-related death and the fifth most common cancer worldwide, with high incidence rates in Eastern Asia, Central and Eastern Europe and South America^1,2^. At diagnosis, approximately 40% of patients have distant metastases (stage IV) with a median overall survival (OS) of approximately 1 year when treated with systemic therapy^3,4^. Spread to the peritoneal cavity is the most common form of dissemination in patients with gastric cancer, and recent population-based studies estimate the incidence of peritoneal metastases at diagnosis to range from 10% to 21%^5^. Advancements in imaging techniques have resulted in an increased documentation of peritoneal metastases. Moreover, a significant variation in incidence exists between surgical and oncological series. This is probably caused by the fact that patients with systemic metastases on CT are often deemed to have unresectable disease and may not undergo staging laparoscopy, contributing to lower incidences reported in oncological series^5^.

Palliative systemic chemotherapy is standard of care for patients with peritoneal metastases of gastric cancer, but its efficacy is hampered by suboptimal drug delivery due to the peritoneal–plasma barrier^6,7^. Moreover, commonly observed symptoms due to peritoneal dissemination such as bowel obstruction and ascites have a negative prognostic value. This is reflected by a median OS of only 9 months after treatment with systemic chemotherapy^8^. Recent investigations have therefore focused on local treatment in the peritoneal cavity to overcome this barrier. In patients with limited isolated peritoneal metastases, cytoreductive surgery combined with a single heated administration of chemotherapy (CRS-HIPEC) is a promising option, but its value remains to be elucidated^6^. In patients with more extensive peritoneal metastases, repeated administration of cytotoxic drugs into the peritoneal cavity has been investigated. Normothermic catheter-based intraperitoneal chemotherapy and normothermic pressurized intraperitoneal aerosol chemotherapy (PIPAC) are methods to deliver intraperitoneal chemotherapy with a palliative intent^9^. In this way, higher intraperitoneal concentrations of cytotoxic drugs can be achieved compared to intravenous administration^9^. For normothermic catheter-based intraperitoneal chemotherapy, an access port is placed subcutaneously and connected to an intraperitoneal catheter. Chemotherapy is administered repeatedly through this port at the outpatient clinic, frequently combined with concomitant systemic chemotherapy (bidirectional chemotherapy)^9^. PIPAC is an intraperitoneal chemotherapy delivery technique using pressurized administered chemotherapy during laparoscopy, which might lead to enhanced uptake and deeper tumour penetration due to the pressurization^9^.

Many studies on intraperitoneal chemotherapy (both normothermic catheter-based intraperitoneal chemotherapy and PIPAC) with a palliative intent in patients with peritoneal metastases of gastric cancer have been performed in the past decade, but sample sizes were often too small to draw definitive conclusions about the efficacy and safety, and all but two were non-randomized trials. To estimate the effectiveness and safety of different methods of intraperitoneal chemotherapy delivery as palliative treatment for patients with peritoneal metastases of gastric origin, we performed a systematic review and meta-analysis of the available literature.

## Methods

### Search strategy and selection criteria

This systematic review and meta-analysis was performed according to the PRISMA guidelines^10^ and registered in PROSPERO, the international prospective register of systemic reviews (PROSPERO registration number: CRD42022375887). A librarian searched Embase, MEDLINE (OvidSP), Web of Science and Cochrane until the 15 January 2024 to identify potentially relevant publications regarding patients with gastric cancer and peritoneal metastases, the use of repeated administration of intraperitoneal chemotherapy with palliative intent, and overall survival. Peritoneal disease was defined as the presence of peritoneal disease of gastric origin in patients who received intraperitoneal chemotherapy as a palliative treatment without planned curatively intended surgical resection.

We excluded studies in which intraperitoneal chemotherapy was part of a curatively intended surgical resection (for example, perioperative or adjuvant) for primarily resectable peritoneal disease, as these patients represent a separate group with a different prognosis and treatment intent. Studies reporting patients who underwent conversion surgery in case of an excellent response to palliatively intended intraperitoneal treatment were not excluded. The strategy included the search terms ‘intraperitoneal chemotherapy’, ‘intraperitoneal drug administration’, ‘pressurized intraperitoneal aerosol chemotherapy’, ‘peritoneum’, ‘stomach’ and relevant variants thereof. No language restrictions or date restrictions were applied. Moreover, we screened the references lists of the included papers to identify additional studies. The *Supplementary Materials* (pp. 2–3) include the full search strategy.

After removal of duplicates, NADG and BJN independently screened the publications based on title and abstract. Case reports, letters to the editor and reviews were excluded. Disagreements were solved by discussion and when eligibility was met, the study proceeded to full-text assessment. Full-text studies were excluded if intraperitoneal chemotherapy was given as part of curatively intended surgery (that is, (neo)adjuvant or perioperative), if the study was a conference abstract without full text, if overall survival was not reported, if the intraperitoneal chemotherapy regimen was not defined or if the language was other than English. If the same patient cohort was presented in another study, the article with the longest follow-up was included.

### Outcome

The primary outcome was overall survival. Secondary outcomes were toxicity and morbidity rate, quality of life when assessed using validated questionnaires, and clinicopathological outcomes of conversion surgery after response to intraperitoneal chemotherapy.

### Data extraction, definitions and quality assessment

A predefined data extraction sheet was used, which included study characteristics (first author, year of publication, country where the study was conducted and study design), study population (total number of patients and median follow-up), definition of peritoneal disease burden (classification for measuring peritoneal disease and the definition for unresectable peritoneal metastases), type of intervention (intraperitoneal regimen, administered intraperitoneal dose, median number of cycles and systemic treatment) and outcomes (median OS in months, 1-year overall survival, proportion of patients who underwent conversion surgery after treatment, grade 3 and 4 adverse events graded according to the Common Terminology Criteria for Adverse Events (CTCAE), surgical complications according to the Clavien–Dindo (CD) classification, quality of life assessment). OS was defined as time from start of intraperitoneal chemotherapy treatment until death. If a control group was present, all aforementioned data for these patients and an HR were collected as well. Moreover, we contacted the authors to obtain the standard error of the median overall survival.

Risk of bias was assessed using the Newcastle–Ottawa scale for non-randomized studies and the Revised Cochrane risk-of-bias tool (RoB-2) for randomized studies^11,12^.

### Statistical analysis

All statistical analyses were performed using R 4.2.1 (R Foundation for Statistical Computing, Vienna, Austria).

A Bayesian random effect model was used to calculate the pooled median OS, as we hypothesized heterogeneity across the studies. For the pooled median OS, we used the median OS adapted from the original publications combined with the standard error retrieved from the corresponding authors. Sensitivity analyses were performed by omitting one study at a time to evaluate the impact of each study on the pooled median OS. To compare median OS between the treatment groups, the Bayesian *P* (*P*_β_) was calculated by computing the probability that the posterior distribution of each group is smaller/larger than the median OS of another group. A separate meta-analysis for the pooled HR of the RCTs was performed by estimating the log HR and the standard error calculated from the published HRs and confidence intervals. Grade 3 or 4 adverse events were calculated as number of events per 100 patients. Publication bias was assessed using a funnel plot and funnel plot asymmetry was tested using Egger’s test. Two-sided *P* less than 0.050 were considered statistically significant.

## Results

### Study and patient characteristics

**Fig. 1:**
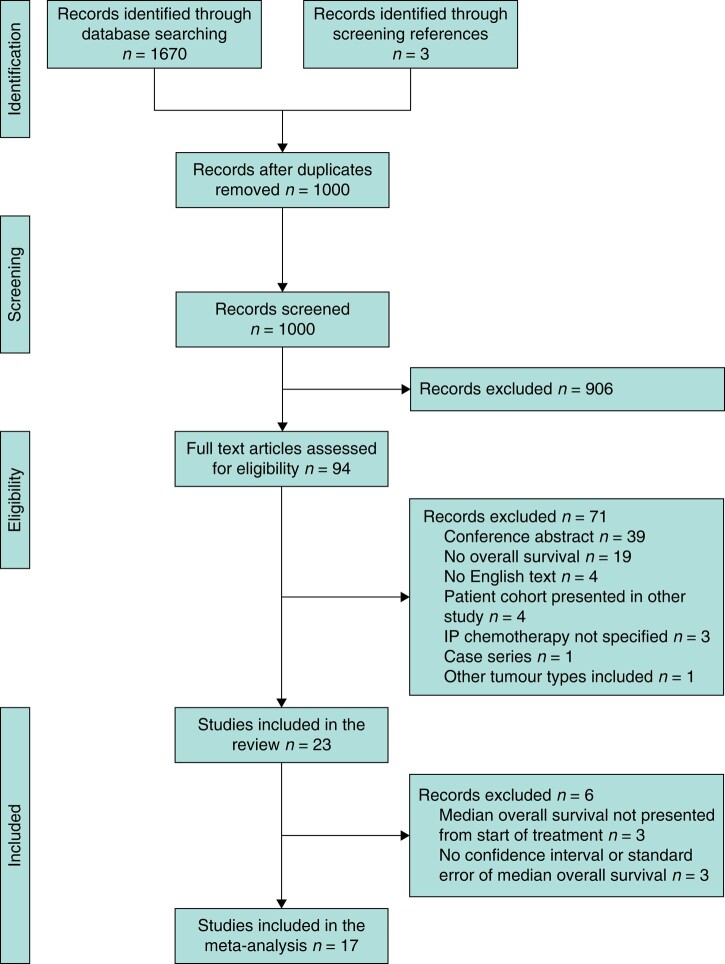
Flow chart of study selection

We identified 1000 potentially relevant publications (*Fig. 1*), of which 23 studies were included in the review and 17 studies in the meta-analysis for overall survival. Six studies were not included in the meta-analysis but only included in the descriptive analyses, as three studies did not present survival data calculated from start of treatment^13–15^, and three of these studies did not report a confidence interval or standard error of the median OS^16–18^.

There were 2 randomized phase III studies^19,20^, 12 non-randomized phase II studies^17,21–31^, 1 prospective feasibility study^13^, 5 analyses of prospective registry databases^14,18,32–34^ and 3 retrospective cohort studies^15,16,35^. No studies investigating HIPEC as stand-alone treatment for unresectable peritoneal disease (that is, without a surgical resection) were identified. *Table S2* shows the study characteristics.

The 23 studies included a total of 999 patients, of whom the median age ranged from 47 to 66 years old. Of all patients, 504 (51%) were men and 495 (49%) were women. Eleven studies were performed in Asian countries (514 patients, 51% of total)^19–24,26–30^. The Japanese Gastric Cancer Association (JGCA) classification and the Peritoneal Cancer Index (PCI) were used for measuring peritoneal disease. The JGCA classification evaluates the presence of positive cytology (C0 or C1) and the presence of macroscopic peritoneal metastases (P0 or P1)^36^, whereas the PCI scores the extent of macroscopic peritoneal disease in 13 pelvic–abdominal regions^37^. Three studies used the JGCA classification^21,27,29^, 14 studies reported the PCI score^13–18,22,25,30–35^ and 5 studies used both methods^19,23,24,26,28^. One study did not report a classification for peritoneal metastases^20^. Seventeen studies defined peritoneal disease as macroscopic peritoneal disease (that is, P1 on JGCA classification or PCI ≥ 1)^13,15–19,22,24–26,28,29,31–35^. The remaining six studies defined peritoneal disease as positive cytology in ascites or macroscopic peritoneal disease^14,20,21,23,27,30^. In 816 patients the peritoneal disease stage was described, of whom the majority (97%) had macroscopic peritoneal disease; 3% of patients had positive cytology only. Ovarian metastases and lymph node metastases (both regional or distant) were most commonly seen as extraperitoneal disease in the studies that allowed metastases other than peritoneal metastases. Extraperitoneal disease was most commonly reported in patients receiving intraperitoneal (i.p.) docetaxel (*n* = 50, 45%), followed by i.p. paclitaxel (*n* = 47, 11%) and PIPAC (*n* = 15, 4%). The disease burden of the patients in each study is further specified in *Table 1*.

**Table 1:** Disease burden of patient population per study

Study	Type of i.p. treatment	Median PCI (range)	Total *N* of patients	Extraperitoneal disease	Prior lines of systemic chemotherapy before i.p. treatment				
					At least 1 prior line (%)	2 lines (%)	3 lines (%)	4 lines (%)	5 lines (%)
Cho^28^	i.p. docetaxel	NR	39	*n* = 17 regional LN (44%), *n* = 5 metastatic LN (13%), *n* = 2 ovary (5%), *n* = 2 pleural effusion (5%)	0	0	0	0	0
Bin^20^	i.p. docetaxel	NR	39	*n* = 24 (62%), not specified	0	0	0	0	0
Lo Dico^13^	i.p. docetaxel	33 (30–39)	6	Ovarian metastases only (*n* = NR)	50	17	0	0	0
Fushida^29^	i.p. docetaxel	NR	27	*n* = 6 LN (22%), *n* = 2 liver (7%), *n* = 1 lung (4%)	0	0	0	0	0
Yamaguchi^26^	i.p. paclitaxel	Not specified†, but 41% between 10 and 19	35	*n* = 4 ovary (24%), *n* = 11 LN (31%)	43	0	0	0	0
Ishigami^21^	i.p. paclitaxel	NR	40	*n* = 16 LN (40%), *n* = 6 ovary (15%)	43	NR	NR	NR	NR
Tu^30^	i.p. paclitaxel	12 (i.q.r.: 4–15)	49	Not permitted	0	0	0	0	0
Ishigami^19^	i.p. paclitaxel	9 (i.q.r.: 4–17)	114	Ovarian metastases only (*n* = NR)	23	NR	NR	NR	NR
Chia^27^	i.p. paclitaxel	NR	44	Not permitted	0	0	0	0	0
Saito^23^	i.p. paclitaxel	14 (0–39)	44	*n* = 2 ovary (5%), *n* = 1 para-aortic LN (2%)	7	NR	NR	NR	NR
Shi^24^	i.p. paclitaxel	Not specified†, but 57% ≥ 20	30	*n* = 7 ovary (23%)	23	NR	NR	NR	NR
Kim^16^	i.p. paclitaxel	22 (±12)*	82	Not permitted	38	0	0	0	0
Kobayashi^22^	i.p. paclitaxel	9 (1–39)	53	Ovarian metastases only (*n* = NR)	0	0	0	0	0
Alyami^33^	PIPAC	17 (1–39)	42	Not permitted	100	47.6	11.9	0	0
Di Giorgio^34^	PIPAC	20 (3–32)	28	*n* = 2 metastatic LN (7%), *n* = 1 liver (4%), *n* = 1 ovary (4%), 1 pleural (4%)	100	14.2	25	0	0
Sindayigaga^14^	PIPAC	15 (1–39)	144	Not permitted	91	30	13	5 (≥4 lines)	
Struller^25^	PIPAC	15 (± 11)*	25	Isolated pleural diffusion only (*n* = NR)	100	64	20	8	8
Gockel^32^	PIPAC	14 (2–36)	24	*n* = 4 liver (17%), *n* = 1 adrenal gland (4%), *n* = 1 pleural (4%)	83	54	16	0	0
Khomyakov^17^	PIPAC	16 (NR)*	31	Regional LN only (*n* = NR)	23	NR	NR	NR	NR
Ellebæk^31^	PIPAC	11 (2–39)	20	Not permitted	95	25	0	0	0
Nadiradze^35^	PIPAC	16 (± 10)*	24	*n* = 3 pleural (13%), *n* = 1 liver (4%)	79	36	17 (≥3 lines)		
Tidadini^15^	PIPAC	18 (12–20)	17	Not permitted	100	NR	NR	NR	NR
Casella^18^	PIPAC	16 (IQR: 8–26)	42	Ovarian metastases only (*n* = NR)	NR	NR	NR	NR	NR

*Mean with standard deviation presented instead of median. †When PCI score was not given as median or mean, the largest group is presented. i.p.: intraperitoneal; LN, lymph node; NR, not reported; PCI, Peritoneal Cancer Index; PIPAC, pressurized intraperitoneal aerosol chemotherapy.

In nine studies, paclitaxel (with doses ranging from 20 to 80 mg/m^2^ 3–5 weekly, *n* = 491) was given as intraperitoneal chemotherapy^16,19,21–24,26,27,30^. Six studies reported the median number of cycles of i.p. paclitaxel, which ranged from 3 to 16 cycles. Four studies administered i.p. docetaxel (doses 30–100 mg/m^2^ 3–4 weekly, *n* = 111)^13,20,28,29^. Of those, three studies reported a median number of 1–8 cycles. The remaining 10 studies used PIPAC (all with cisplatin 7.5 mg/m^2^ and doxorubicin 1.5 mg/m^2^, *n* = 397)^14,15,17,18,25,31–35^. The median number of cycles with PIPAC in seven studies reporting was 2–3 cycles. Concomitant systemic therapy was administered to all patients in 19 studies (*n* = 796)^15–24,26–30,32–34^. In three studies a subset of patients received systemic therapy (*n =* 188, of whom 39 received systemic therapy)^14,31,35^ and in one study no concomitant systemic therapy was given (*n* = 25)^25^ (*Table S1*). In 12 studies, the type of systemic therapy was specified. Oxaliplatin and fluorouracil or a derivate thereof were used in the majority of studies (9 studies, 278 patients); paclitaxel and S1 was administered in two studies (154 patients), cisplatin and capecitabine in one study (39 patients), cisplatin and S1 in one study (53 patients) and S-1 monotherapy in one study (27 patients).

Most patients who underwent PIPAC had also been treated with systemic chemotherapy before inclusion in the study. In three studies on i.p. docetaxel, all patients were chemonaive (total patients treated with i.p. docetaxel that not received prior treatment: 105, 95% of total)^20,28,29^. Two studies on i.p. paclitaxel did not permit prior chemotherapy, resulting in a total of 93 chemonaive patients (21% of total patients with i.p. paclitaxel)^27,30^.

Two of the included studies were randomized phase III trials, and both were with catheter-based intraperitoneal chemotherapy^19,20^. The first study administered i.p. paclitaxel 20 mg/m^2^ on days 1 and 8 in 3-week cycles, concomitant with S-1 and systemic paclitaxel^19^. The other study used i.p. docetaxel 30 mg/m^2^ on days 1 and 8, in combination with S-1 and oxaliplatin^20^.

### Quality and risk of bias assessment

According to the Newcastle–Ottawa Scale (NOS), 7 non-randomized studies were graded as moderate risk of bias^13,14,17,25,31,33,35^ and the remaining 14 non-randomized studies as low risk of bias^15,16,18,21–24,26–28,30,32,34^ (*Table S3*). With the RoB-2 tool, one randomized study had a moderate risk of bias^19^, whereas for the other randomized study the risk of bias was low^20^ (*Table S4*). The funnel plot for studies included in the meta-analysis is presented in *Fig. S1*. Egger’s test revealed no evidence for asymmetry (*P* = 0.13), indicating no publication bias.

### Overall survival

**Fig. 2:**
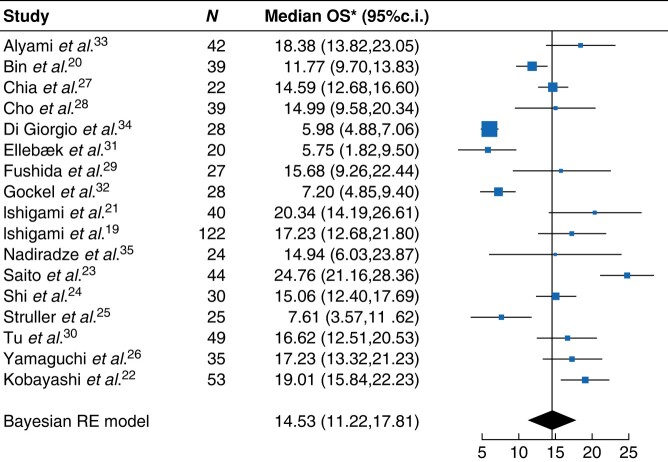
Forest plot for pooled median overall survival for all included studies *Median overall survival data per study are calculated using a random-effects (RE) model and therefore slightly differ from *Table 2*. Standard deviation of RE model = 6.1.

Median OS since the start of intraperitoneal therapy ranged from 4.7 months to 25.8 months (*Table S5*). Pooled median OS was 14.5 months (95%c.i.: 11.2 to 17.8 months) for all intraperitoneal chemotherapy treatments (*Fig. 2*). The pooled median OS for i.p. docetaxel, i.p. paclitaxel and PIPAC was 13.2 months (95%c.i.: 3.6 to 25.1 months), 18.4 months (95%c.i.: 15.0 to 22.2 months) and 9.0 months (95%c.i.: 2.3 to 16.5 months) respectively (*Fig. S2*). Median OS of i.p. paclitaxel was significantly higher compared to PIPAC (*P*_β_ = 0.001). The median OS of i.p. paclitaxel was not significantly higher than the median OS of intraperitoneal docetaxel (*P*_β_ = 0.111). The median OS of intraperitoneal docetaxel did also not significantly differ from the median OS of PIPAC (*P*_β_ = 0.090). The sensitivity analyses showed stable results. A sub-analysis with studies that only included patients with isolated, macroscopic peritoneal disease (3 catheter-based i.p., 2 PIPAC) did not show a statistical difference in median OS (17.2 months for catheter-based i.p., 11.9 months for PIPAC, *P*_β_ = 0.284)^19,22,24,31,33^. The pooled HR of the two RCTs was 0.64 (95%c.i.: 0.47 to 0.86) in favour of the intraperitoneal chemotherapy arm (*Fig. S3*).

Eleven studies reported 1-year OS^13,15,17,21–23,26,27,29,30,35^. In six studies on i.p. paclitaxel, 1-year OS ranged from 67.8% to 81.6%. In three studies on PIPAC 1-year OS was 49.8–94.1%, and the remaining two studies on i.p. docetaxel reported a 1-year OS of 67.0–70.4% (*Table S5*).

### Toxicity and morbidity rate

Nineteen studies reported CTCAE grade 3 or 4 adverse events, representing a total of 837 patients (83.8%). Two studies did not present toxicity data^15,32^ and two studies did not grade the reported toxicity^16,18^. A total of 690 grade 3 or 4 adverse events were seen (82 events per 100 patients, *Table S6*). The most common adverse events were neutropenia (20 events per 100 patients), leukopenia (10 events per 100 patients) and anaemia (80 events per 100 patients). Adverse event rates were similar for both drugs used for catheter-based intraperitoneal chemotherapy, with i.p. paclitaxel reporting 127 events per 100 patients and i.p. docetaxel 122 events per 100 patients. For studies on PIPAC, 10 events per 100 patients were reported. Four studies reported postoperative complications after PIPAC according to the Clavien–Dindo classification, resulting in five reported major complications (CD grade ≥III) in 129 patients (4%)^15,18,32,34^. The postoperative complications consisted of small bowel perforation (CD grade III), obstructive jaundice (CD grade IIIb), haemorrhagic shock (CD grade IV) and two times a recurrence of ascites (CD grade IIIIa).

Nine deaths potentially related to intraperitoneal chemotherapy treatment were reported in four studies (1.0% of patients with toxicity data available)^14,22,27,33,35^. Three deaths occurred in patients receiving i.p. paclitaxel (two neutropenic sepsis, one peritonitis due to tumour perforation) and six cases occurred in patients receiving PIPAC, all within 30 days after the most recent PIPAC treatment. Causes of death were ileus (*n* = 3), ascites decompensation (*n* = 1), pulmonary embolism (*n* = 1) and cardiac failure (*n* = 1).

### Quality of life

Quality of life (QOL) data were reported in two prospective studies on PIPAC (*n* = 34), using the EORTC QLQ-C30 questionnaire^25,32^. The first study found no statistically significant difference in functional and symptom score before and after the first PIPAC treatment, although an impairment of >10 points was seen for diarrhoea and pain (+11.9 and 15.5 points respectively)^25^. The second study reported a stable global health score and a slightly decreased functioning and symptom scores after receiving two PIPAC treatments^32^.

### Conversion surgery

The proportion of patients who underwent conversion surgery after completion of intraperitoneal chemotherapy ranged from 0% to 60%. In five studies, no data on conversion surgery were reported (*Table 2*)^16,19,20,32,35^. In another six studies, the rate of R0 resection (primary tumour and biopsied scar-like areas on peritoneal surface) was reported, and ranged between 21% and 100%^18,23,24,27,29,30^. When pooling all studies, a total of 163 conversion surgeries were performed in 999 patients (16%). In 78 cases the resection margin was described, of which an R0 resection margin was reported in 55 patients (71%). The most common procedures were total gastrectomy in eight studies, followed by CRS-HIPEC in six studies. Twenty-three postoperative complications (12%) were reported after conversion surgery (*Table 2*). Postoperative anastomotic leakage was most commonly reported (*n* = 7), followed by fistula (pancreatic fistula *n* = 3, intestinal fistula *n* = 1 and oesophago-jejunal fistula *n* = 1) and thoracenteses (*n* = 3). The median OS of patients that underwent conversion surgery ranged from 24 to 42 months. A sensitivity analysis with only including studies in which conversion surgery was not performed in any patient resulted in a pooled median OS of 11.3 months (95%c.i.: 4.7 to 18.1 months) for intraperitoneal chemotherapy^19,20,28,31,32,35^.

**Table 2:** Proportion of patients that underwent conversion surgery

Study	Number of patients	Type of i.p. treatment	Conversion surgery	R0 resection	Type of resection	Survival after resection	Postoperative complications
Cho^28^	39	i.p. docetaxel	0 (0%)	NA	NA	NA	NA
Bin^20^	39	i.p. docetaxel	NR	NA	NA	NA	NA
Lo Dico^13^	6	i.p. docetaxel	1 (17%)	NR	CRS-HIPEC	mOS: not reached (alive at cut-off)	None
Fushida^29^	27	i.p. docetaxel	14 (52%)	3 (21%)	Radical gastrectomy and removal of peritoneal deposit site	NR	Pancreatic fistula (*n* = 3), anastomotic leakage (*n* = 1), CD grade in both not specified
Yamaguchi^26^	35	i.p. paclitaxel	21 (60%)	NR	Radical gastrectomy	NR	NR
Ishigami^21^	40	i.p. paclitaxel	16 (40%)	NR	Radical gastrectomy	NR	NR
Tu^30^	49	i.p. paclitaxel	9 (18%)	9 (100%)	Radical gastrectomy	mOS: 33.4 months (95%c.i. 30.7–36.1)	Intestinal fistula (CD grade not specified, *n* = 1)
Ishigami^19^	114	i.p. paclitaxel	NR	NA	NA	NA	NA
Chia^27^	44	i.p. paclitaxel	13 (30%)	9 (69%)	Radical gastrectomy	mOS: 24.2 months	Delayed bleeding (CD grade IIIB, *n* = 1) and duodenal stump leak (CD grade IIIB, *n* = 1), chyle leak and intra-abdominal collections (CD grade IIIA, *n* = 4)
Saito^23^	44	i.p. paclitaxel	20 (44%)	14 (70%)	Radical gastrectomy	mOS not reached, 1 year OS: 100% (95%c.i. 69.5–100%)	Leakage (CD grade II, *n* = 1)
Shi^24^	30	i.p. paclitaxel	11 (37%)	11 (100%)	Radical gastrectomy	mOS: 24.6 months	Abdominal infection (CD grade not specified, *n* = 1)
Kim^16^	82	PIPAC	NR	NA	NA	NA	NA
Kobayashi^22^	53	i.p. paclitaxel	26 (30%)	NR	Radical gastrectomy	mOS: 42.1 months (95%c.i. 34.9–43.5)	NR
Alyami^33^	42	PIPAC	6 (14%)	NR	CRS-HIPEC	NR	NR
Di Giorgio^34^	28	PIPAC	1 (4%)	NR	CRS-HIPEC	mOS: not reached (alive at cut-off)	None
Sindayigaga^14^	144	PIPAC	10 (7%)	NR	CRS-HIPEC	NR	NR
Struller^25^	25	PIPAC	2 (8%)	NR	CRS-HIPEC	NR	NR
Gockel^32^	24	PIPAC	NR	NA	NA	NA	NA
Khomyakov^17^	31	PIPAC	0 (0%)	NA	NA	NA	NA
Ellebæk^31^	20	PIPAC	0 (0%)	NA	NA	NA	NA
Nadiradze^35^	24	PIPAC	NR	NA	NA	NA	NA
Tidadini^15^	17	PIPAC	2 (12%)	NR	NR	NR	NR
Casella^18^	42	PIPAC	11 (26%)	9 (82%)	CRS + HIPEC (in 7 patients)	NR	Thoracentesis (CD grade IIIa, *n* = 3), oesophago-jejunal fistula (CD grade IIIa, *n* = 1), pneumothorax (CD grade IIIa, *n* = 1), 5 additional CD grade 1/2 complications
Total	999		163 (16%)	55 (71%)*			23 (14%)

*Percentage calculated with only the studies that reported the R0 resection rate. CD, Clavien–Dindo classification; CRS-HIPECc, cytoreductive surgery + hyperthermic intraperitoneal chemotherapy; i.p., intraperitoneal; mOS, median overall survival; NA, not applicable; NR, not reported; PIPAC, pressurized intraperitoneal aerosol chemotherapy.

## Discussion

This meta-analysis including 17 studies on intraperitoneal chemotherapy with palliative intent for peritoneal metastases of gastric cancer found a pooled median OS of 14.5 months (95%c.i.: 11.2 to 17.8 months) since start of intraperitoneal treatment and, based on two RCTs, an HR of 0.64 in favour of bidirectional chemotherapy compared to systemic chemotherapy only. Furthermore, patients treated with i.p. paclitaxel had a significantly higher median OS compared to patients treated with PIPAC, whereas fewer grade 3 or 4 adverse events were reported in patients who underwent PIPAC.

Several reviews have focused on intraperitoneal chemotherapy in combination with a surgical resection with curative intent, but pooled data on the effect of intraperitoneal chemotherapy in the palliative setting are lacking. The pooled median OS of 14.5 months is promising in the light of the median OS of 9–11 months reported with systemic chemotherapy only^8,38,39^. Although the favourable pooled survival might be partly explained by patient selection, the survival benefit found in the meta-analysis of the two randomized trials strengthens the suggestion that intraperitoneal chemotherapy improves oncological outcome.

No studies directly comparing normothermic catheter-based i.p. chemotherapy and PIPAC have been performed yet, but this meta-analysis found a small survival benefit of i.p. paclitaxel compared to PIPAC. These results should, however, be carefully and critically interpreted, as heterogeneity between groups hampers direct comparison. A sub-analysis on a homogeneous group of patients (isolated macroscopic peritoneal disease) did not find a statistically significant difference in survival between normothermic catheter-based i.p. chemotherapy and PIPAC, but was hampered by a small sample size. Prior treatment with systemic chemotherapy was more common in the PIPAC group compared to patients who had i.p. paclitaxel or docetaxel. Moreover, it is unknown if this effect is caused by differences in application method or type of chemotherapy. Although the optimal timing of intraperitoneal treatment is not yet known, it is presumably more effective in chemonaive patients, as better outcomes are expected prior to development of resistance to the systemic chemotherapy. Conversely, by selecting patients who are in condition for PIPAC after systemic therapy for PIPAC, a selection of a patient subgroup with a better prognosis and possibly more chemo-sensitive disease is made. Taking into account the heavily pretreated status of the patients receiving PIPAC, the pooled median OS of 9.0 months is encouraging.

Moreover, the incidence and location of extraperitoneal disease differed among the studies. Excluding patients with extraperitoneal disease would hamper the external validity as approximately 35% of the patients with synchronous peritoneal metastases have other distant metastases^8^. Further studies will need to compare the different intraperitoneal treatment methods and regimens, and examine which patients benefit most from intraperitoneal treatment.

Morbidity rate and toxicity seem limited. In all studies, nine deaths (1.0%) were potentially related to intraperitoneal chemotherapy, of which most often occurred due to postoperative complications in the PIPAC group (6/9 reported deaths). Pooled rates of grade 3 or 4 adverse events were higher in the i.p. paclitaxel and docetaxel groups compared to PIPAC. Unfortunately, we could not correct the rate of adverse events for the time on treatment, as these data were not reported in most studies. Furthermore, the lack of patient-level data prevented determining whether grade 3 or 4 adverse events clustered within individual patients, thereby hindering the calculation of the exact percentage of patients experiencing such events. In patients who had i.p. paclitaxel and docetaxel treatment, haematological adverse events were most commonly observed. These adverse events were presumably attributed to the concomitantly administered systemic chemotherapy. Interestingly, no haematological adverse events were described in the studies on PIPAC, possibly due to the fact that PIPAC is most often sequenced with systemic chemotherapy instead of given concomitantly. Of note, these were primarily retrospective studies, which may have led to under-reporting of the actual adverse events. In 2020, the randomized phase II EstoK 01 trial (NCT04065139) was started, comparing PIPAC + systemic chemotherapy *versus* systemic chemotherapy alone. This study was stopped prematurely due to an unexpected and yet unexplained high number of deaths in the PIPAC arm. This emphasizes the need for careful assessment of the toxicity and feasibility of PIPAC in controlled circumstances.

QOL assessment was performed in 34 of 999 patients only, all receiving PIPAC treatment, and did not show a severe deterioration of quality of life. As these treatments are mostly applied in the palliative setting, incorporating QOL assessment is utterly important in future studies on intraperitoneal chemotherapy.

Although normothermic catheter-based intraperitoneal chemotherapy and PIPAC are both primarily treatments with a palliative intent, in 16% (ranging between 0% and 60%) of patients conversion surgery (either radical gastrectomy or CRS-HIPEC) after completion of intraperitoneal chemotherapy treatment was performed. There was substantial heterogeneity across the studies in the proportion of patients undergoing conversion surgery, which could be explained by differences in peritoneal load and by the fact that conversion surgery was not a primary aim in most studies. Furthermore, there is no consensus on the role for gastrectomy or CRS-HIPEC after response to intraperitoneal chemotherapy^40^. R0 resection was achieved in 71% of the cases and median OS varied substantially between studies, emphasizing the need for further (randomized) trials on the additional value of surgery in patients with a response to peritoneal treatment. Future studies should determine which subgroups of patients might benefit from conversion surgery. Factors that could be important for selection of potential candidates for conversion surgery include extent of peritoneal disease at baseline, response to chemotherapy and performance status.

The main limitation of this meta-analysis is the heterogeneity in patient selection between the studies, both in extent of peritoneal disease and systemic chemotherapy pretreatment. Most studies on i.p. paclitaxel and docetaxel were prospectively performed and inclusion criteria were clearly described, but this was not the case for all PIPAC studies. Moreover, most studies on i.p. paclitaxel and docetaxel were performed in Asia, whereas most studies on PIPAC were from European centres. Differences in tumour biology, screening programmes and treatment strategies exist between countries and further limit comparison of outcomes^41^. Second, studies used different methods to classify peritoneal disease (either PCI or JGCA classification) and definitions of peritoneal disease not eligible for curative treatment. The majority of patients had macroscopic peritoneal disease (97%), but in the few studies using the JGCA classification the amount of macroscopic peritoneal disease was not clearly described. Consensus on the preferred classification system and the definition of incurable peritoneal disease would ease the comparison of future studies. Finally, this meta-analysis only reports OS as an oncological outcome measure as this was most commonly reported. Ideally, quantifying peritoneal response would give more insight in the effect of intraperitoneal chemotherapy. However, the optimal imaging modality to assess peritoneal response had not yet been determined. Conventional CT scan has moderate sensitivity for detection of peritoneal metastases, whereas other methods such as MRI, FAPI PET/CT scan or liquid biopsy techniques such as ct-DNA measurement might be better modalities to detect peritoneal metastases and evaluate treatment^42^. However, as these modalities were not incorporated in most studies, progression-free survival could not be calculated accurately.

## Supplementary Material

znae116_Supplementary_Data

## Data Availability

Data can be made available by the authors on justified request. Part of the results of this study were presented during the annual congress of the European Society for Medical Oncology (ESMO, Madrid, 20–24 October 2023; abstract number: 1583P; *Annals of Oncology* 34:S882, DOI: https://doi.org/10.1016/j.annonc.2023.09.1495).
