# Supplementary material for: Intraperitoneal chemotherapy for peritoneal metastases of gastric origin: a systematic review and meta-analysis
Source: Br J Surg. 2024 May 9;111(5):znae116. doi: 10.1093/bjs/znae116 (PMC11081074; doi:10.1093/bjs/znae116)

**Intraperitoneal chemotherapy for peritoneal metastases of gastric origin: a systematic review and meta-analysis**

Niels A.D. Guchelaar^1^, Kazem Nasserinejad^2,3^, Bianca Mostert^1^, Stijn L.W. Koolen^1,4^, Pieter C. van der Sluis^5^, Sjoerd M. Lagarde^5^, Bas P.L. Wijnhoven^5^, Ron H.J. Mathijssen^5^, Bo J. Noordman^5^

^1^ Department of Medical Oncology, Erasmus Medical Center Cancer Institute, Rotterdam, the Netherlands.

^2^ Department of Hematology, Erasmus Medical Center Cancer Institute, Rotterdam, the Netherlands.

^3^ HOVON Foundation, Rotterdam, the Netherlands.

^4^ Department of Pharmacy, Erasmus Medical Center, Rotterdam, the Netherlands.

^5^ Department of Surgery, Division of Surgical Oncology and Gastrointestinal Surgery, Erasmus Medical Center Cancer Institute, Rotterdam, the Netherlands.

**Corresponding author.** Niels A.D. Guchelaar, Department of Medical Oncology, Erasmus MC Cancer Institute, Dr. Molewaterplein 40, 3015 GD, Rotterdam, The Netherlands, T: 0031107039640, E: n.guchelaar@erasmusmc.nl, ORCID: 0000-0001-8080-9093

**Supplementary Materials - Index**

| **Supplementary Methods** |  |
| --- | --- |
| Detailed search strategy | *page 3* |
| **Supplementary Figures and Tables** |  |
| Table S1: Articles retrieved from different databases | *page 4* |
| Table S2: Study characteristics | *pages 5 - 7* |
| Table S3: Quality assessment of non-randomized studies included with the Newcastle-Ottawa Scale (NOS) | *page 8* |
| Table S4: Evaluation of quality of randomized studies included with the Revised Cochrane risk-of-bias tool (RoB-2) | *page 8* |
| Table S5: Median overall survival since start treatment and 1-year overall survival | *page 9* |
| Table S6: Grade 3 or 4 adverse events | *pages 10 - 11* |
| Figure S1: Funnel plot | *page 12* |
| Figure S2: Forest plot divided per type of intraperitoneal chemotherapy | *page 13* |
| Figure S3: Forest plot showing the association between treatment and overall survival in the included randomized controlled trials (intraperitoneal + systemic chemotherapy versus systemic chemotherapy only) | *page 14* |

**Supplementary Methods**

**Detailed search strategy**

**embase.com**

('intraperitoneal chemotherapy'/de OR (('cancer chemotherapy'/exp OR 'antineoplastic agent'/mj/exp OR cisplatin/de OR paclitaxel/de OR irinotecan/de) AND 'intraperitoneal drug administration'/de) OR 'antineoplastic agent'/mj/exp/dd_ip OR 'pressurized intraperitoneal aerosol chemotherapy'/de OR (((intraperiton* OR intra-periton*) NEAR/3 (chemotherap* OR antineoplastic* OR anti¬-neoplastic* OR cisplatin* OR paclitaxel* OR irinotecan* OR docetaxel*)) OR PIPAC):Ab,ti,kw) AND ('peritoneum cancer'/exp OR 'peritoneal disease'/de OR (((periton* OR intraperiton*) NEAR/3 (cancer OR Metasta*))):Ab,ti,kw) AND ('stomach tumor'/exp OR ((gastric* OR stomach*) NEAR/6 (cancer* OR tumor* OR tumour* OR neoplas* OR carcinoma* OR adenocarcinoma*)):Ab,ti,kw) NOT (thermotherapy/mj OR (thermotherap* OR Hypertherm* OR cytoreduct* OR cyto-reduct* OR hipec):ti) NOT ([animals]/lim NOT [humans]/lim)

**Medline ALL**

(((exp *Antineoplastic Agents/ OR Cisplatin/ OR Paclitaxel/ OR Irinotecan/) AND exp Injections, Intraperitoneal/) OR (((intraperiton* OR intra-periton*) ADJ3 (chemotherap* OR antineoplastic* OR anti-neoplastic* OR cisplatin* OR paclitaxel* OR irinotecan* OR docetaxel*)) OR PIPAC).ab,ti,kf.) AND (exp Peritoneal Neoplasms/ OR (((periton*) ADJ3 (cancer OR Metasta* OR mesotheliom*))).ab,ti,kf.) NOT (* Cytoreduction Surgical Procedures/ OR (thermotherap* OR Hypertherm* OR cytoreduct* OR cyto-reduct* OR hipec).ti.) AND (Stomach Neoplasms / OR ((gastric* OR stomach*) ADJ6 (cancer* OR tumor* OR tumour* OR neoplas* OR carcinoma* OR adenocarcinoma*)).ab,ti,kw.) NOT (*Hyperthermia, Induced/ OR (thermotherap* OR Hypertherm* OR cytoreduct* OR cyto-reduct* OR hipec).ti.) NOT (exp animals/ NOT humans/)

**Web of Science Core Collection**

TS=((((intraperiton* OR intra-periton*) NEAR/2 (chemotherap* OR antineoplastic* OR anti-neoplastic* OR cisplatin* OR paclitaxel* OR irinotecan* OR docetaxel*)) OR PIPAC) AND ((((periton*) NEAR/2 (cancer OR Metasta* OR mesotheliom*)))) AND (((gastric* OR stomach*) NEAR/5 (cancer* OR tumor* OR tumour* OR neoplas* OR carcinoma* OR adenocarcinoma*))) NOT ((thermotherap* OR Hypertherm* OR cytoreduct* OR cyto-reduct* OR hipec) ) NOT ((animal* OR rat OR rats OR mouse OR mice OR murine OR dog OR dogs OR canine OR cat OR cats OR feline OR rabbit OR cow OR cows OR bovine OR rodent* OR sheep OR ovine OR pig OR swine OR porcine OR veterinar* OR chick* OR zebrafish* OR baboon* OR nonhuman* OR primate* OR cattle* OR goose OR geese OR duck OR macaque* OR avian* OR bird* OR fish*) NOT (human* OR patient* OR women OR woman OR men OR man)))

**Cochrane Central Register of Controlled Trials**

((((intraperiton* OR intra NEXT periton*) NEAR/3 (chemotherap* OR antineoplastic* OR anti NEXT neoplastic* OR cisplatin* OR paclitaxel* OR irinotecan* OR docetaxel*)) OR PIPAC):Ab,ti) AND ((((periton*) NEAR/3 (cancer OR Metasta* OR mesotheliom*))):Ab,ti) AND (((gastric* OR stomach*) NEAR/6 (cancer* OR tumor* OR tumour* OR neoplas* OR carcinoma* OR adenocarcinoma*)):Ab,ti,kw) NOT ((thermotherap* OR Hypertherm* OR cytoreduct* OR cyto NEXT reduct* OR hipec):ti)

**Supplementary Figures and Tables**

**Table S1: Articles retrieved from different databases.**

| Database searched | via | Years of coverage | Records | Records after duplicates removed |
| --- | --- | --- | --- | --- |
| Medline ALL | Ovid | 1946 - Present | 430 | 428 |
| Embase | Embase.com | 1971 - Present | 867 | 498 |
| Web of Science Core Collection* | Web of Knowledge | 1975 - Present | 286 | 73 |
| Cochrane Central Register of Controlled Trials | Wiley | 1992 - Present | 87 | 1 |
| Through reference lists screening |  |  | 3 | 3 |
| Total | | | **1673** | **1000** |

*Science Citation Index Expanded (1975-present) ; Social Sciences Citation Index (1975-present) ; Arts & Humanities Citation Index (1975-present) ; Conference Proceedings Citation Index- Science (1990-present) ; Conference Proceedings Citation Index- Social Science & Humanities (1990-present) ; Emerging Sources Citation Index (2015-present) ** Manually deleted abstracts from trial registries.

|  |  |  |  |  |  |  |  |  | |  |  |  |
| --- | --- | --- | --- | --- | --- | --- | --- | --- | --- | --- | --- | --- |
| Type of study | **Author, year** | **Country** | **Period of inclusion** | **Total N of patients** | **Median age (years; range)** | **Classification system** | **Definition unres. disease** | **Peritoneal disease stage** | | **Intraperitoneal treatment** | **Median number of IP cycles** | **Systemic treatment** |
|  |  |  |  |  |  |  |  |  | |  |  |  |
|  |  |  |  |  |  |  |  | **Macroscopic** | **Positive cytology only** |  |  |  |
| Phase III, randomised | Ishigami, 2018[19] | Japan | 2011-13 | 114 | 60 (25-74) | JGCA and PCI | Pos. histology | 114 (100%) | .. | Paclitaxel (20 mg/m^2^) at day 1 and 8 in 3-week cycles | Not reported | Paclitaxel + S-1 |
|  | Bin, 2022[20] | China | 2016-18 | 39 | 51 (33-68) | Not reported | Pos. histology and/or cytology | Not reported | Not reported | Docetaxel (30 mg/m^2^) at day 1 and 8 in 3-week cycles | Not reported | Oxaliplatin + S-1 |
| Phase II, single arm | Ishigami, 2010[21] | Japan | 2006-07 | 40 | 62 (29-86) | JGCA | Pos. histology and/or cytology | 34 (85%) | 6 (15%) | Paclitaxel (20 mg/m^2^) at day 1 and 8 in 3-week cycles | 7 (range 1-23) | Paclitaxel + S-1 |
|  | Kobayashi, 2024 [22] | Japan | 2017-18 | 53 | 61 (23-74) | PCI | Pos. histology | 53 (100%) |  | Paclitaxel (20 mg/m^2^) at day 1, 8 and 22 in 5-week cycles | 7 (range 1-34) | Cisplatin + S-1 |
|  | Saito, 2021[23] | Japan | 2016-19 | 44 | 64 (37-77) | JGCA and PCI | Pos. histology and/or cytology | 42 (95%) | 2 (5%) | Paclitaxel (40 mg/m^2^) at day 1 and 8 in 3-week cycles | 16 (range 1-48) | Oxaliplatin + S-1 |
|  | Shi, 2021[24] | China | 2017-19 | 30 | 51 (29-74) | JGCA and PCI | Pos. histology | 30 (100%) | .. | Paclitaxel (40 mg/m^2^) at day 1 and 8 in 3-week cycles | 6 (range: 2-16) | Oxaliplatin + S-1 |
|  | Struller, 2019[25] | Germany | 2013-17 | 25 | Mean (± SD):  55 ± 13^*^ | PCI | Pos. histology | 25 (100%) | .. | PIPAC (cisplatin 7.5 mg/m^2^ and doxorubicin 1.5 mg/m^2^) in 6-week cycles | 2 (range: 1-3) | None |
|  | Yamaguchi, 2013[26] | Japan | 2009-10 | 35 | 55 (28-74) | JGCA and PCI | Pos. histology | 35 (100%) | .. | Paclitaxel (20 mg/m2) at day 1 and 8 in 3-week cycles | 11 (range: 3-35) | Oxaliplatin + S-1 |
|  | Chia, 2022[27] | Singapore | 2013-19 | 44 | Mean (± SD):  61 ± 9^*^ | JGCA | Pos. histology and/or cytology | 41 (93%) | 3 (7%) | Paclitaxel (40 mg/m2) at day 1 and 8 in 3-week cycles | Not reported | Oxaliplatin + capecitabine |
|  | Cho, 2017[28] | Korea | 2011-13 | 39 | 49 (31–73) | JGCA and PCI | Pos. histology | 39 (100%) | .. | Docetaxel (100 mg/m^2^) at day 1 in 3-week cycles | 8 (range: 1-8) | Cisplatin + capecitabine |
|  | Fushida, 2013[29] | Japan | 2007-10 | 27 | 66 (26-75) | JGCA | Pos. histology | 27 (100%) | .. | Docetaxel (45 mg/m^2^) at day 1 and 15 in 4-week cycles | 4 (range: 2-11) | S-1 |
|  | Tu, 2022[30] | China | 2017-19 | 49 | 55 (30-78) | PCI | Pos. histology and/or cytology | 35 (71%) | 14 (29%) | Paclitaxel (80 mg/m^2^) at day 1 in 3-week cycles | 3 | Oxaliplatin + S-1 |
|  | Khomyakov, 2016[17] | Russia | 2013-16 | 31 | Mean: 52^*^ | PCI | Pos. histology | 31 (100%) | .. | PIPAC (cisplatin 7.5 mg/m^2^ and doxorubicin 1.5 mg/m^2^) in 6-week cycles | Not reported | Oxaliplatin + capecitabine |
|  | Ellebæk, 2020[31] | Denmark | 2015-18 | 20 | 59 (31-70) | PCI | Pos. histology | 20 (100%) | .. | PIPAC (cisplatin 7.5 mg/m^2^ and doxorubicin 1.5 mg/m^2^) in 6-week cycles | Not reported | In 9 patients (type not specified) |
| Prospective exploratory | Lo Dico, 2020[13] | France | 2014-16 | 6 | 47 (24-66) | PCI | Pos. histology | 6 (100%) | .. | Docetaxel (30 mg/m^2^) at day 1, 8 and 15 in 4-week cycles | 1 (range: 1-2) | Oxaliplatin + 5-fluoruracil |
| Prospective registry | Gockel, 2018[32] | Germany | 2015-18 | 24 | 57 (44-75) | PCI | Pos. histology | 24 (100%) | .. | PIPAC (cisplatin 7.5 mg/m^2^ and doxorubicin 1.5 mg/m^2^) in 6-week cycles | 2 (range: 1-6) | In all patients (type not specified) |
|  | Alyami, 2021[33] | France | Not reported | 42 | 52 (32-75) | PCI | Pos. histology | 42 (100%) | .. | PIPAC (cisplatin 7.5 mg/m2 and doxorubicin 1.5 mg/m2) in 6-week cycles | 3 (range: 1-12) | In all patients (type not specified) |
|  | Di Giorgio, 2020[34] | Italy | 2017-19 | 28 | 50 (38-79) | PCI | Pos. histology | 28 (100%) | .. | PIPAC (cisplatin 7.5 mg/m2 and doxorubicin 1.5 mg/m2) in 6 to 8-week cycles | Not reported | In all patients (several regimens) |
|  | Sindayigaga, 2022[14] | Germany | Not reported | 144 | 57 (22-88) | PCI | Pos. histology and/or cytology | Not reported | Not reported | PIPAC (cisplatin 7.5 mg/m2 and doxorubicin 1.5 mg/m2) in 6-week cycles | 3 (range: 1-3) | In 22 patients (type not specified) |
|  | Casella, 2023 [18] | Italy | 2019-22 | 42 | 61 | PCI | Pos. histology | 42 (100%) |  | PIPAC (cisplatin 7.5 mg/m2 and doxorubicin 1.5 mg/m2) in 6-week cycles | Not reported | In all patients (several regimens) |
| Retrospective | Nadiradze, 2016[35] | Germany | Not reported | 24 | Mean (± SD):  56 ± 13^*^ | PCI | Pos. histology | 24 (100%) | .. | PIPAC (cisplatin 7.5 mg/m2 and doxorubicin 1.5 mg/m2) in 6-week cycles | 2 (range: 1-5) | In 8 patients (type not specified) |
|  | Tidadini, 2022[15] | France | 2016-20 | 17 | 64 | PCI | Pos. histology | 17 (100%) | .. | PIPAC (cisplatin 7.5 mg/m2 and doxorubicin 1.5 mg/m2) in 6-week cycles | 2 | In all patients (several regimens) |
|  | Kim, 2020[16] | South Korea | 2015-18 | 82 | 56 (25-82) | PCI | Pos. histology | 82 (100%) | .. | Paclitaxel (20-80 mg/m^2^) at day 1 in 3-week cycles | Not reported | In all patients (several regimens) |
| Total | .. | .. | .. | 999 | .. | .. | .. | 791 (97%)† | 25 (3%)† | .. |  | .. |

**Table S2: Study characteristics.**

Definition unres. disease clarifies whether positive peritoneal histology, or positive peritoneal cytology, or both were required to fulfill the definition of unresectable peritoneal disease. * = Median age was not presented so therefore the mean (± SD) was taken. † = Percentage of the number of patients of whom peritoneal disease stage was available. *Abbreviations:* JGCA: Japanese Gastric Cancer Association classification; N: number of patients; PCI: Peritoneal Cancer Index; PIPAC: pressurized intraperitoneal aerosol chemotherapy; pos; positive; unres: unresectable.

**Table S3: Quality assessment of non-randomized studies included with the Newcastle-Ottawa Scale (NOS)**

|  | Selection | | | | Comparability | | Outcome | | |  |
| --- | --- | --- | --- | --- | --- | --- | --- | --- | --- | --- |
| Study | **Repre-sentative of the exposed cohort** | **Selec-tion of external control** | **Ascertain-ment of exposure** | **Outcome of interest not present at start study** | **Comparability of cohorts** | | **Assessment of outcomes** | **Sufficient follow-up time ^a^** | **Adequacy of follow-up** | **Total (9/9)** |
|  |  |  |  |  | **Main factor** | **Additional factor** |  |  |  |  |
| Ishigami, 2010 | * | 0 | * | * | * | 0 | * | * | * | 7/9 |
| Saito, 2021 | * | 0 | * | * | * | 0 | * | * | * | 7/9 |
| Shi, 2021 | * | 0 | * | * | * | 0 | * | * | * | 7/9 |
| Struller, 2019 | 0 | 0 | * | * | * | 0 | * | * | 0 | 5/9 |
| Yamaguchi, 2013 | * | 0 | * | * | * | 0 | * | * | * | 7/9 |
| Chia, 2022 | * | * | * | * | * | * | * | * | * | 9/9 |
| Cho, 2017 | * | 0 | * | * | * | 0 | * | * | * | 7/9 |
| Fushida, 2013 | * | 0 | * | * | * | 0 | * | * | * | 7/9 |
| Gockel, 2018 | * | 0 | * | * | * | 0 | * | * | * | 7/9 |
| Alyami, 2021 | * | 0 | * | * | * | 0 | 0 | 0 | * | 5/9 |
| Di Giorgio, 2020 | * | 0 | * | * | * | 0 | * | * | * | 7/9 |
| Sindayigaga, 2022 | * | 0 | * | * | * | 0 | * | 0 | * | 6/9 |
| Nadiradze, 2016 | * | 0 | * | * | * | 0 | * | 0 | * | 6/9 |
| Lo Dico, 2020 | 0 | 0 | * | * | * | 0 | * | * | * | 6/9 |
| Tu, 2022 | * | 0 | * | * | * | 0 | * | * | * | 7/9 |
| Tidadini, 2022 | * | 0 | * | * | * | 0 | * | * | * | 7/9 |
| Khomyakov, 2016 | * | 0 | * | * | * | 0 | * | * | 0 | 6/9 |
| Ellebæk, 2020 | * | 0 | * | * | * | 0 | * | 0 | * | 6/9 |
| Kim, 2020 | * | 0 | * | * | * | 0 | * | * | * | 7/9 |
| Kobayashi, 2024 | * | 0 | * | * | * | 0 | * | * | * | 7/9 |
| Casella, 2023 | * | 0 | * | * | * | 0 | * | * | * | 7/9 |

*^a^  One year of follow-up was chosen to be enough for an event to occur.[8]*

*The NOS tool uses a star system to judge a study on three perspectives: selection, comparability and outcome. Studies scoring 7 to 9, 4 to 6, and 0 to 3 are regarded as low risk of bias, some concerns of bias, and high risk of bias, respectively.*

**Table S4: Evaluation of quality of randomized studies included with the Revised Cochrane risk-of-bias tool (RoB-2)**

| Study | Randomization process | Deviations from intended interventions | Missing outcome data | Measurement of the outcome | Selection of the reported result | Overall |
| --- | --- | --- | --- | --- | --- | --- |
| Ishigami, 2018 |  |  |  |  |  |  |
| Bin, 2022 |  |  |  |  |  |  |

*The RoB-2 tool reports five risk of bias domains. Green color represents low risk of bias, yellow some concerns, and red high risk of bias.*

**Table S5: Median overall survival since start treatment and 1-year overall survival**

| Study | Number of patients | Type of IP treatment | Median overall survival since start treatment (months; 95% CI) | 1-year overall survival (%; 95% CI) | Median progression-free survival (months; 95% CI) |
| --- | --- | --- | --- | --- | --- |
| Cho, 2017 | 39 | IP docetaxel | 15.1 (9.1-21.1) | Not reported | 11.0 (6.9-15.1) |
| Bin, 2022 | 39 | IP docetaxel | 11.7 (9.6-13.8) | Not reported | Not reported |
| Lo Dico, 2020 | 6 | IP docetaxel | Not reported | 67 (not reported) | Not reported |
| Fushida, 2013 | 27 | IP docetaxel | 16.2 (8.4-22.1) | 70.4 (53.2-87.4) | Not reported |
| Yamaguchi, 2013 | 35 | IP paclitaxel | 17.6 (13.4-22.3) | 77.1 (60.5-88.1) | Not reported |
| Ishigami, 2010 | 40 | IP paclitaxel | 22.5 (16.6-32.9) | 78.0 (65.0-90.0) | Not reported |
| Tu, 2022 | 49 | IP paclitaxel | 16.9 (13.6-20.2) | 81.6 (68.6-90.0) | 6.5 (2.9-10.1) |
| Ishigami, 2018 | 114 | IP paclitaxel | 17.7 (12.8-21.8) | Not reported | Not reported |
| Chia, 2022 | 44 | IP paclitaxel | 14.6 (12.6-16.6) | 67.8 (not reported) | 9.5 (8.3-10.8) |
| Saito, 2021 | 44 | IP paclitaxel | 25.8 (16.3-27.6) | 79.5 (64.4-88.8) | 18.2 (not reported) |
| Shi, 2021 | 30 | IP paclitaxel | 15.1 (12.4-17.8) | Not reported | 6.6 (4.7-8.5) |
| Kim, 2020 | 82 | IP paclitaxel | Not reported | Not reported | Not reported |
| Kobayashi, 2024 | 53 | IP paclitaxel | 19.4 (16.1-24.6) | 73.6 (59.5-83.4) | 11.1 (8.4-15.9) |
| Alyami, 2021 | 42 | PIPAC | 19.1 (14.1-25.3) | Not reported | Not reported |
| Di Giorgio, 2020 | 28 | PIPAC | Not reported | Not reported | Not reported |
| Sindayigaga, 2022 | 144 | PIPAC | Not reported | Not reported | Not reported |
| Struller, 2019 | 25 | PIPAC | 6.7 (2.5-12.0) | Not reported | Not reported |
| Gockel, 2018 | 24 | PIPAC | 6.9 (3.5-11.7) | Not reported | Not reported |
| Khomyakov. 2016 | 31 | PIPAC | Not reported | 49.8 (not reported) | Not reported |
| Ellebæk, 2020 | 20 | PIPAC | 4.7 (0.7-15.6) | Not reported | Not reported |
| Nadiradze, 2016 | 24 | PIPAC | 15.4 (2.8-28.0) | 52 (not reported) | Not reported |
| Tidadini, 2022 | 17 | PIPAC | 12.8 (7.2-34.3) | 94.1 (65.0-99.2) | Not reported |
| Casella, 2023 | 42 | PIPAC | Not reported | Not reported | Not reported |

*Abbreviations:* IP: intraperitoneal; PIPAC: pressurized intraperitoneal aerosol chemotherapy.

|  | Cho[28] | Bin[20] | Lo Dico[13] | Fushida[29] | Yamaguchi[26] | Ishigami[21] | Tu[30] | Ishigami[19] | Chia[27] | Saito[23] |  | Shi[24] | Kobayashi[22] | Alyami[33] | Di Giorgio[34] | Nadiradze[35] | Sindayigaga[14] | Struller[25] | Ellebæk[31] | Khomyakov[16] | Total (events per 100 patients) |
| --- | --- | --- | --- | --- | --- | --- | --- | --- | --- | --- | --- | --- | --- | --- | --- | --- | --- | --- | --- | --- | --- |
| Type of IP therapy | Doce | Doce | Doce | Doce | Pacli | Pacli | Pacli | Pacli | Pacli | Pacli |  | Pacli | Pacli | PIPAC | PIPAC | PIPAC | PIPAC | PIPAC | PIPAC | PIPAC |  |
| Number of patients | 39 | 39 | 6 | 27 | 35 | 40 | 49 | 116 | 44 | 44 |  | 30 | 53 | 42 | 28 | 25 | 144 | 25 | 20 | 31 | 837 |
| Grade 3 or 4 events | 84 | 40 | 2 | 9 | 26 | 32 | 76 | 183 | 33 | 42 |  | 51 | 80 | 5 | 2 | 12 | 7 | 3 | 2 | 1 | 690 (82) |
| Neutropenia | 15 | 10 | .. | 2 | 12 | 15 | 20 | 58 | .. | 17 |  | 7 | 13 | .. | .. | .. | .. | .. | .. | .. | 169 (20) |
| Leucopenia | 2 | 8 | 1 | 2 | 8 | 7 | 9 | 29 | .. | 5 |  | 7 | 4 | .. | .. | .. | .. | .. | .. | .. | 82 (10) |
| Anemia | 10 | 5 | .. | .. | 3 | 4 | 11 | 15 | 1 | 7 |  | 5 | 16 | .. | .. | .. | .. | .. | .. | .. | 77 (9) |
| Anorexia | 5 | 4 | .. | 5 | .. | 3 | 4 | 12 | 1 | .. |  | .. | 9 | .. | .. | .. | .. | .. | .. | .. | 42 (5) |
| Diarrhea | 3 | 2 | .. | .. | 1 | 1 | 6 | 10 | 3 | 1 |  | 6 | 7 | .. | .. | .. | .. | .. | .. | .. | 40 (5) |
| Vomiting | 5 | 2 | .. | .. | 1 | 3 | 4 | 4 | .. | 1 |  | 8 | 1 | .. | .. | .. | .. | .. | .. | .. | 29 (3) |
| Fatigue | 8 | 2 | 1 | .. | 1 | .. | 3 | 9 | 1 | 1 |  | .. | 3 | .. | .. | .. | .. | .. | .. | .. | 29 (3) |
| Febrile neutropenia | 9 | 1 | .. | .. | .. | .. | .. | 9 | 4 | 2 |  | .. | 2 | .. | .. | .. | .. | .. | .. | .. | 27 (3) |
| Neuropathy | 4 | .. | .. | .. | .. | .. | 2 | 2 | 1 | 1 |  | 12 | .. | .. | .. | .. | .. | .. | .. | .. | 22 (3) |
| Nausea | 2 | 2 | .. | .. | .. | .. | 7 | 8 | .. | .. |  | .. | 3 | .. | .. | .. | .. | .. | .. | .. | 22 (3) |
| Abdominal pain | 12 | .. | .. | .. | .. | .. | 4 | .. | 1 | .. |  | .. | 1 | .. | .. | 2 | .. | 1 | .. | .. | 21 (3) |
| Hypokalemia | .. | .. | .. | .. | .. | .. | .. | 8 | 6 | .. |  | .. | 6 | .. | .. | .. | .. | .. | .. | .. | 20 (2) |
| Elevated ALT and AST | .. | 2 | .. | .. | .. | .. | .. | 8 | .. | .. |  | .. | 1 | .. | 2 | 5 | .. | .. | .. | .. | 18 (2) |
| Thrombocytopenia | .. | 2 | .. | .. | .. | .. | 3 | .. | 1 | 1 |  | 6 | 2 | .. | .. | .. | .. | .. | .. | .. | 15 (2) |
| Hyponatremia | .. | .. | .. | .. | .. | .. | .. | 4 | 1 | .. |  | .. | 6 | .. | .. | .. | .. | .. | .. | .. | 11 (1) |
| Port infection | 2 | .. | .. | .. | .. | .. | .. | .. | 4 | 3 |  | .. | .. | .. | .. | .. | .. | .. | .. | .. | 9 (1) |
| Oral mucositis | 4 | .. | .. | .. | .. | .. | .. | 2 | .. | .. |  | .. | 1 | .. | .. | .. | .. | .. | .. | .. | 7 (1) |
| Fever | .. | .. | .. | .. | .. | .. | .. | 4 | 2 | .. |  | .. | .. | .. | .. | .. | .. | .. | .. | .. | 6 (1) |
| Peritonitis | .. | .. | .. | .. | .. | .. | .. | .. | 1 | .. |  | .. | .. | .. | .. | 3 | .. | .. | .. | .. | 4 (1) |
| Hand-foot syndrome | 2 | .. | .. | .. | .. | .. | .. | .. | 1 | .. |  | .. | .. | .. | .. | .. | .. | .. | .. | .. | 3 (<1) |
| Alopecia | .. | .. | .. | .. | .. | .. | 3 | .. | .. | .. |  | .. | .. | .. | .. | .. | .. | .. | .. | .. | 3 (<1) |
| Port obstruction | .. | .. | .. | .. | .. | .. | .. | .. | .. | 3 |  | .. | .. | .. | .. | .. | .. | .. | .. | .. | 3 (<1) |
| Allergic reaction | .. | .. | .. | .. | .. | .. | .. | .. | .. | .. |  | .. | .. | 2 | .. | 1 | .. | .. | .. | .. | 3 (<1) |
| Creatinine increase | .. | .. | .. | .. | .. | .. | .. | 1 | .. | .. |  | .. | 2 | .. | .. | .. | .. | .. | .. | .. | 3 (<1) |
| Ileus | .. | .. | .. | .. | .. | .. | .. | .. | .. | .. |  | .. | .. | 1 | .. | .. | .. | 1 | 1 | .. | 3 (<1) |
| UTI | .. | .. | .. | .. | .. | .. | .. | .. | 2 | .. |  | .. | .. | .. | .. | .. | .. | .. | .. | .. | 2 (<1) |
| Constipation | 1 | .. | .. | .. | .. | .. | .. | .. | .. | .. |  | .. | .. | .. | .. | .. | .. | .. | .. | .. | 1 (<1) |
| Tumor perforation | .. | .. | .. | .. | .. | .. | .. | .. | 1 | .. |  | .. | .. | .. | .. | .. | .. | .. | .. | .. | 1 (<1) |
| Hypotension | .. | .. | .. | .. | .. | .. | .. | .. | 1 | .. |  | .. | .. | .. | .. | .. | .. | .. | .. | .. | 1 (<1) |
| Pulmonary embolism | .. | .. | .. | .. | .. | .. | .. | .. | .. | .. |  | .. | .. | 1 | .. | .. | .. | .. | .. | .. | 1 (<1) |
| Capnothorax | .. | .. | .. | .. | .. | .. | .. | .. | .. | .. |  | .. | .. | .. | .. | .. | .. | .. | .. | 1 | 1 (<1) |
| Wound dehiscence | .. | .. | .. | .. | .. | .. | .. | .. | .. | .. |  | .. | .. | .. | .. | .. | .. | .. | 1 | .. | 1 (<1) |

**Table S6: Grade 3 or 4 adverse events.**

Cells are empty when the study did not report on that specific adverse event. Abbreviations: ALS: alanine aminotransferase; AST: aspartate aminotransferase; Doce: Intraperitoneal docetaxel; Pacli: Intraperitoneal paclitaxel; PIPAC: Pressurized intraperitoneal aerosol chemotherapy; UTI: urinary tract infection.

**Figure S1: Funnel plot.**


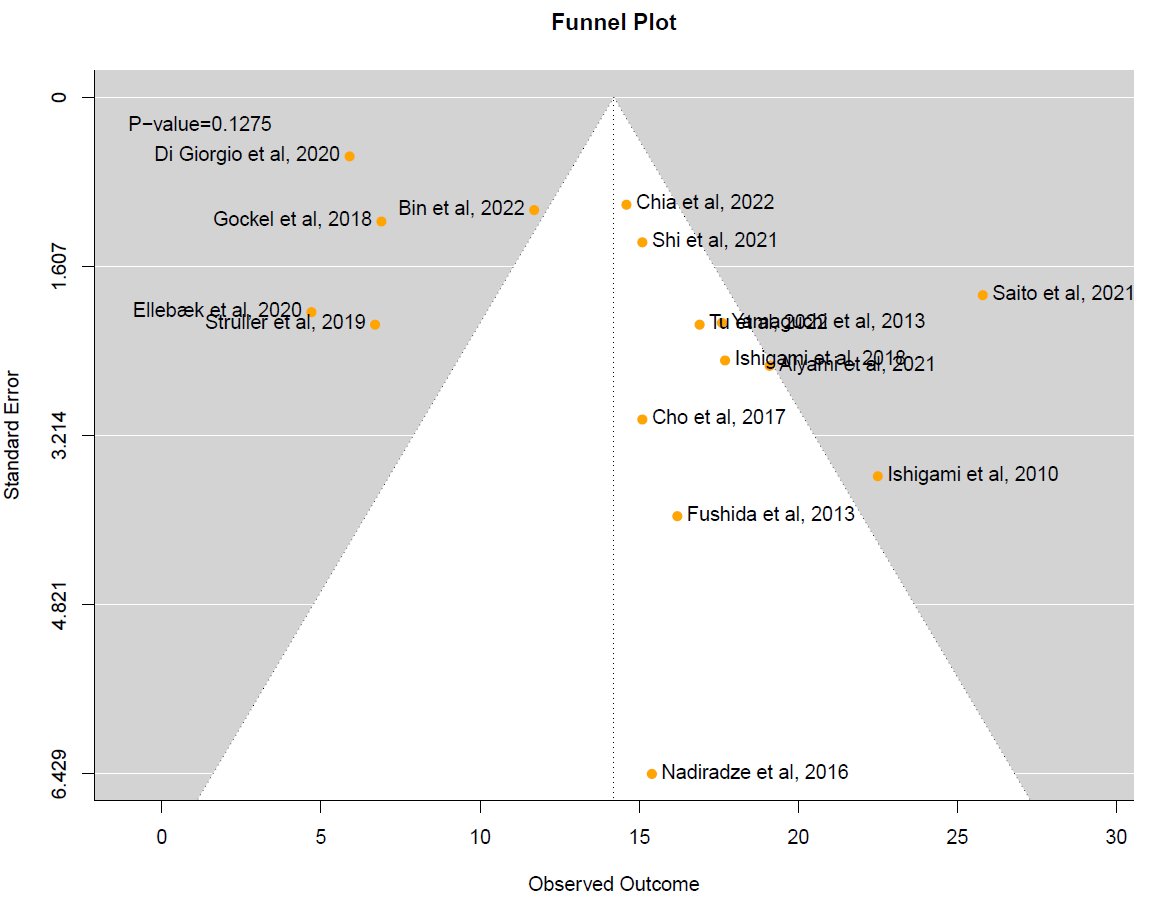


**Figure S2: Forest plot divided per type of intraperitoneal chemotherapy.**

**
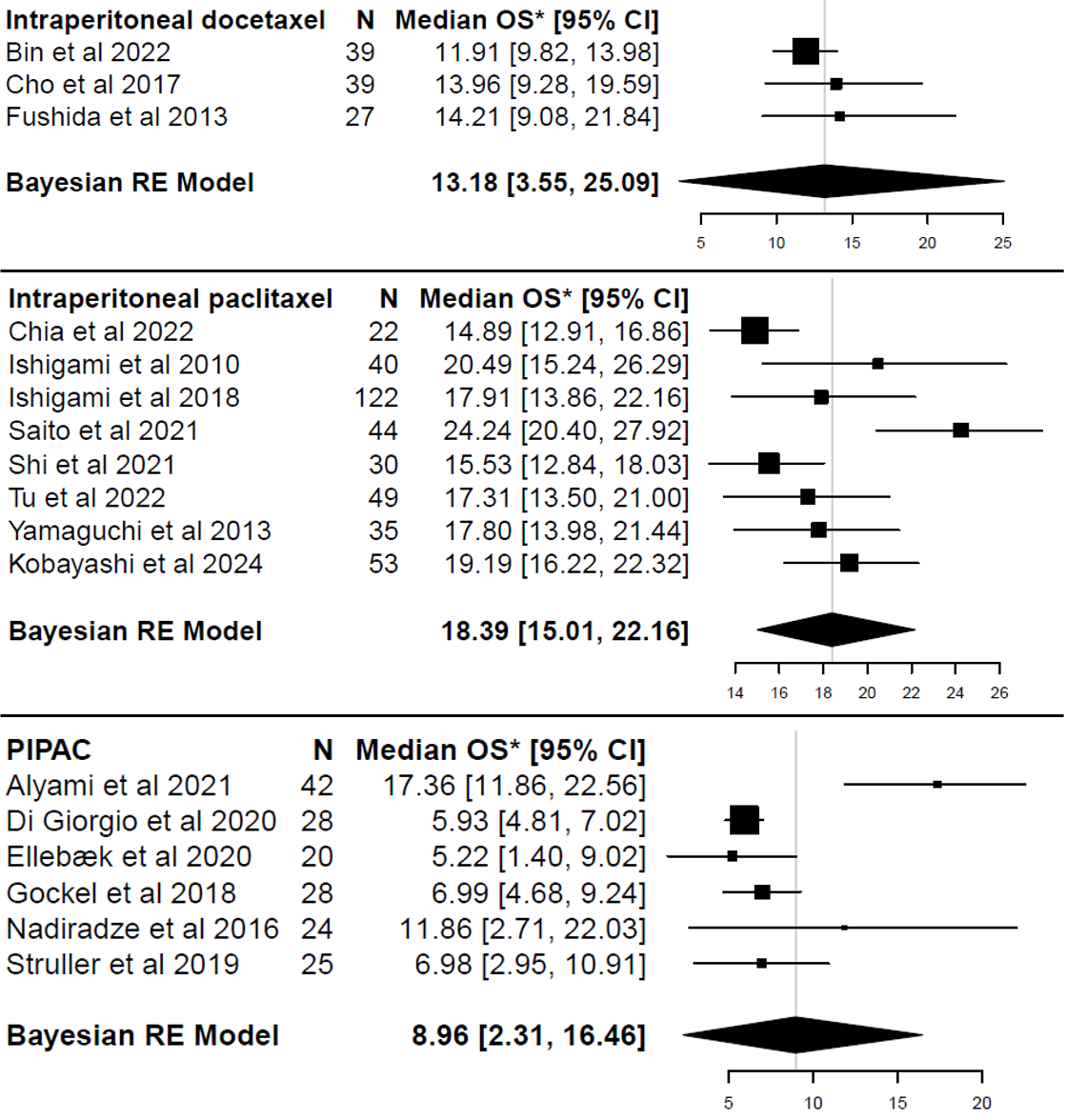
**

*Median overall survival data per study are calculated using a random-effects model and therefore slightly differ from table 2. *Abbreviations:* PIPAC: pressurized intraperitoneal aerosol chemotherapy.

**Figure S3: Forest plot showing the association between treatment and overall survival in the included randomized controlled trials (intraperitoneal + systemic chemotherapy versus systemic chemotherapy only).**


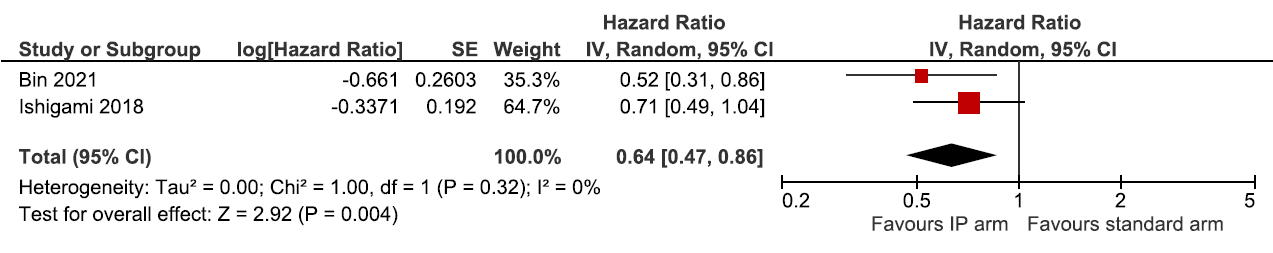

Supplement: znae116_Supplementary_Data [file znae116_supplementary_data.zip › supplementary materials.docx]
